# Pain trajectories of dorsomedial prefrontal intermittent theta burst stimulation versus sham treatment in depression

**DOI:** 10.1186/s12883-020-01881-3

**Published:** 2020-08-20

**Authors:** Erika Malm, Wiebke Struckmann, Jonas Persson, Robert Bodén

**Affiliations:** Department of Neuroscience, Psychiatry, Uppsala University Hospital, Entrance 10, Ground floor, SE-751 85 Uppsala, Sweden

**Keywords:** Repetitive transcranial magnetic stimulation, Scalp pain, Side-effect, Depressive disorder

## Abstract

**Background:**

Prefrontal repetitive transcranial magnetic stimulation is an established add-on treatment for major depressive disorder and is increasingly feasible with protocols of short duration, such as intermittent theta burst stimulation (iTBS). The most common and limiting side effect is pain at the site of application. Our objective was to investigate how pain develops over time in patients with depression receiving iTBS compared to sham stimulation.

**Methods:**

This is a subsample from a randomized clinical trial. Patients received daily sessions of 2400 pulses of dorsomedial prefrontal iTBS or sham stimulation with transcutaneous electric stimulation during 2 to 3 weeks. After unmasking of treatment allocation, patients receiving sham treatment were offered active iTBS in an open phase. Patients rated pain on a scale from 0 to 10 after the last train of stimulation on the first, fifth and final treatment day. A Mann-Whitney U-test was conducted to test for group differences and related-samples Friedman’s tests to analyze changes in pain ratings over time.

**Results:**

The scalp pain in the group receiving iTBS was rated higher than sham treatment on the first (U = 263.5, *p* = 0.035) and fifth day (U = 271.0, *p* = 0.020) but not on the final day (U = 210.5, *p* = 0.121). The pain decreased mainly during the first 5 days of treatment (*χ*^2^ = 0.875, *p* = 0.040). In the open phase the pain decreased from the first day to the final day (*χ*^2^ = 1.194, *p* = 0.001).

**Conclusions:**

The subjective pain perception of active dorsomedial iTBS was higher than sham treatment but decreased over time, indicating an analgesic effect, or habituation. The result from this study can be used to inform patients about what to expect regarding pain during an iTBS treatment course.

**Trial registration:**

Clinicaltrials.gov, NCT02905604. Registered 19 September 2016.

## Background

Repetitive transcranial magnetic stimulation (rTMS) of the dorsolateral prefrontal cortex (DLPFC) is considered an established add-on alternative for treatment resistant depressive disorders and there is also evidence for an analgesic effect when delivered over the primary motor cortex contralateral to the pain [1].

Apart from the DLPFC, new targets for depression treatment have been under investigation, such as the dorsomedial prefrontal cortex (DMPFC) [2].

When targeting the left DLPFC or DMPFC in depression, theta burst stimulation (TBS) is delivered intermittently, in 2 s trains delivered at 10 s intervals. This is believed to increase excitability in the targeted region similar to the traditional high frequency (10 Hz) protocols but with a shorter duration, just over 3 min, compared to almost 38 min [3, 4].

RTMS is an overall well tolerated treatment with scalp pain or discomfort on the application site being the most common issue [5]. In the largest rTMS trial so far, which sought to investigate effectiveness of iTBS versus 10 Hz rTMS, about two-thirds of the patients reported head-ache in both treatment arms – however, the time to reach target intensity was longer in the iTBS arm, indicating longer time to tolerability [4]. A large randomized, sham-controlled trial of 10 Hz rTMS over the left DLPFC showed that the painfulness of active treatment decreased over time, but not during sham treatment with transcutaneous electrical nerve stimulation (TENS) [6]. Whether the pain decreases in a similar way during treatment with iTBS of the DMPFC has to our knowledge not yet been investigated.

The aim of this study was to investigate and compare the pain perception of active and sham iTBS of the DMPFC in patients with depression. The main objective was to study how the pain develops over time and if any differences could be observed between patients receiving active or sham treatment.

## Method

This study includes a subset of patients from a double-blind randomized clinical trial at Uppsala University Hospital, Unit for brain stimulation, investigating the effect of iTBS over the DMPFC on negative symptoms. Recruitment of the patients in this subset with depression was from September 29 in 2016 to October 1 in 2018.

### Subjects

Patients were recruited from the academic psychiatric clinic in Uppsala, Sweden, and enrolled by author RB. For the present subset we only included the patients with a depressive disorder diagnosis verified through a Mini International Neuropsychiatric Interview version 6.0 (M.I.N.I.) [7]. Further inclusion criteria comprised a score of less than 40 points on the Motivation and Pleasure self-rating scale [8], and being between 18 and 59 years old. Concomitant antidepressant medication was allowed but had to be unchanged for the past month, and throughout the study. Exclusion criteria were epilepsy, implants with ferromagnetic material within 30 cm of the treatment coil, implants controlled by physiological signals including pacemakers, implantable cardioverter-defibrillators, vagus nerve stimulators, wearable cardioverter-defibrillators, implanted mediation pumps and intracardiac lines. Other exclusion criteria were active substance use disorder and pregnancy.

### Procedures

The first visit was a screening visit where patients were investigated and scheduled for a baseline visit if they fulfilled all inclusion criteria. The baseline visit included structured psychiatric symptom interviews, self-rating of depressive symptoms with the Montgomery Asberg Depression Rating Scale (MADRS-S) [9], a neurological exam, and evaluation of cortical excitability to obtain the resting motor threshold (RMT). The RMT was obtained by delivery of magnetic pulses over the hot-spot in the medial motor cortex for the extensor hallucis longus, which was localized according to a standardized procedure [3]. The lowest intensity needed to elicit a muscle contraction 50% of the time in the foot (measured as observed contraction), is the RMT, and was estimated using an automated maximum likelihood strategy [10, 11].

The stimulator operating nurse received a randomization code in an envelope prepared by a contract research organization, which was entered in the rTMS machine to obtain correct treatment allocation. Sham or active treatment was given daily during 10–15 weekdays depending on the patients’ ability to reach the target intensity, which was 90% of the individual RMT. During each session at least 50% of the trains had to reach the predetermined treatment intensity to be regarded as a complete treatment session. If a session did not meet these criteria the treatment course was prolonged with 1 day at a time, with a maximum of 15 days for the whole treatment course.

The day after the final treatment, the psychiatric and neurophysiological examinations made on the baseline visit were repeated. The operator and patients were unmasked 4 weeks after baseline. During the open phase, patients allocated in the sham group received active iTBS treatment with the same titration procedure and treatment protocol as described above.

### Treatment protocol

Treatment was applied to the DMPFC which was located with an MRI-based neuronavigational system (TMS Navigator, Localite, Bonn, Germany). The brain target used for navigation was defined as the coordinates x = 0, y = 30, z = 30 in Montreal Neurological Institution (MNI) [12]. The magnetic stimulator used in the trial was the Magpro X100 with Magoption with a Cool-DB80 A/P, which is a combined active/placebo coil with two identical sides. The placebo side of the coil is insulated to prevent approximately 95% of the magnetic field from reaching the patient [13]. The coil has a built-in position sensor which is used by a research software which determined whether the active or placebo side was to be angled towards the patient, depending on the randomization code entered by the operator.

To the active group, rTMS with theta-burst frequency was applied at 90% of resting motor threshold, 80 trains of stimulation (2 s on, 8 s off), each train consisting of ten bursts at 5 Hz and each burst consisting of three biphasic pulses at 50 Hz, a total of 2400 pulses/session.

In the group allocated to sham treatment the magnetic stimulator automatically prompted the user to place the insulated side of the coil towards the patient. For all patients, two TENS electrodes were placed medially on the forehead under the coil. However only in the sham group, patients received a mild TENS with a maximum current of 4 mA, synchronous with the magnetic pulses and scaled to magnetic stimulation intensity to mimic the sensation of active treatment. No TENS was given in the active group. The TENS application is a built-in software within the TMS device, which is activated depending on the entered randomization code, enabling blinding of the operating nurse.

#### Pain rating

Patients were asked to rate the pain they were experiencing directly after the last train of stimulation. Patients decided for themselves if they preferred pain rating with Visual Analogue Scale (VAS) or Numeric Rating Scale (NRS), on a scale from 0 to 10, where 0 is no pain and 10 is worst imaginable pain.

#### Titration

During the first treatments the intensity was individually titrated up aiming to reach 90% of RMT. In the sham group the TENS intensity was automatically scaled to the magnetic stimulation intensity by the built-in software within the TMS device, which enabled titration to reach a TENS intensity corresponding to 90% of RMT. The titration was employed to reduce the discomfort by letting the patients get used to the sensation of the stimulation. The intensity was slowly increased if the patient found the pain manageable, i.e. not rating the pain higher than seven. When at least 50% of the trains reached the predetermined treatment intensity (90% of RMT) the session was regarded as a complete session. The number of days to reach the first complete session was defined as *days to target*.

### Statistical analysis

Shapiro-Wilks test of normality implied that the pain rating data was not normally distributed. Since the data was skewed and pain is measured in ordinal scale, median was chosen as central tendency measure and non-parametric tests were chosen for the analysis.

Baseline demographic and clinical data were assessed with Mann-Whitney U-tests (age, MADRS-S scores) or Chi-square tests (sex, level of education, and primary diagnosis).

To account for the fact that patients received different degrees of stimulation intensity due to variations in titration rate and RMT (which in turn affected the target stimulator intensity), an adjusted pain score was created by dividing the original pain score by actual magnetic stimulator intensity at train 80.

First, a Mann-Whitney U-test was conducted to test for group differences in pain rating on the first, fifth, and final day. Second, a related-samples Friedman’s test of differences among repeated measures was conducted in order to analyze changes of the pain scores over time. Dunn-Bonferroni post hoc tests were carried out, with significance values adjusted by the Bonferroni correction for multiple tests. Finally, the pain score from the open phase was analyzed with a related-samples Friedman’s test of differences among repeated measures and Dunn-Bonferroni post hoc tests.

To explore if treatment response might explain the differences between the two treatment groups a non-parametric ANCOVA was conducted with treatment day as repeated measures and treatment arm as between-groups effect. The covariate was the difference in total MADRS-S rating scores between baseline and follow-up.

A second Mann-Whitney U-test was conducted to test for groups differences in days to target intensity due to titration.

Additional analysis were conducted without adjusting the pain score for stimulator intensity in the titration phase as described above.

Statistical analyzes were conducted IBM SPSS Statistics 25.0.

## Results

A total of 42 patients with depression were included in the study and 36 completed the blind phase. Two declined before randomization, and 21 were allocated to sham and 19 to active iTBS. Three dropped out during the blind phase, two in the sham group between fifth and final day, one due to a serious adverse event, and the other due to poor subjective effect, one in the active group before the fifth day due to practical issues to attend the treatments, and one patient in the active group had missing data on pain rating on the first treatment day. Consequently, for the first and fifth treatment day, 18 patients receiving active and 21 patients receiving sham treatment were available for analysis. Thereafter, one patient in the active group was excluded from analysis of the final day due to unblinding of operator and patient because of faulty magnetic stimulator. Thus, for the final treatment day data was analyzed on 17 patients receiving active and 19 receiving sham treatment. Two of the 21 patients from the sham group did not enter the open phase. One of the remaining 19 patients dropped out before the fifth treatment day.

There were no differences between groups in baseline demographic and clinical characteristics (Table 1). Both groups had a mean MADRS-S score equivalent to a level of moderate depression at baseline (Table 1). After the blind treatment phase, the MADRS-S score was reduced with a mean (SD) of 5.4 (8.5) points in the active group, and with 2.1 (6.3) points in the sham group. The change in depressive symptoms did not differ significantly between the treatment groups (U = 1.38, *p* = 0.175). These MADRS-S change scores have been reported elsewhere [14].

**Table 1:** Baseline demographic and clinical data

	Sham	Active	Test for difference
Sex, N (female/male)	21 (10/11)	19 (10/9)	*p* = 1.000
Age, mean (SD^a^)	29 (9)	30 (10)	*p* = 0.695
Level of education			*p* = 0.129
Not completed year 0–9	1	0	
Completed year 0–9	5	1	
Completed year 10–12	12	10	
Higher education	3	8	
MADRS-S^b^ before treatment, mean (SD)^c^	30 (8)	30 (9)	*p* = 0.989
Primary diagnosis			*p* = 0.380
Bipolar affective disorder^d^	3	1	
Depressive episode/disorder	18	18	
Recurrent depressive disorder	6	9	

^a^Standard deviation

^b^Montgomery Asberg Depression Rating Scale

^c^*N* = 36, who completed the blind treatment phase

^d^Of which 1 was diagnosed bipolar 1

### Titration

Due to titration, there was a trend towards the group allocated to sham treatment reaching target intensity faster (median days to target = 1, IQR = 1), compared to the group receiving active treatment (median days to target = 2, IQR = 4). However, there was no statistically significant group difference (U = 270.5, *p* = 0.054). Median (IQR) treatment duration was 11 (2) days in the active group, and 10 (5) days in the sham group.

### Pain rating

#### Blind phase

**Fig. 1:**
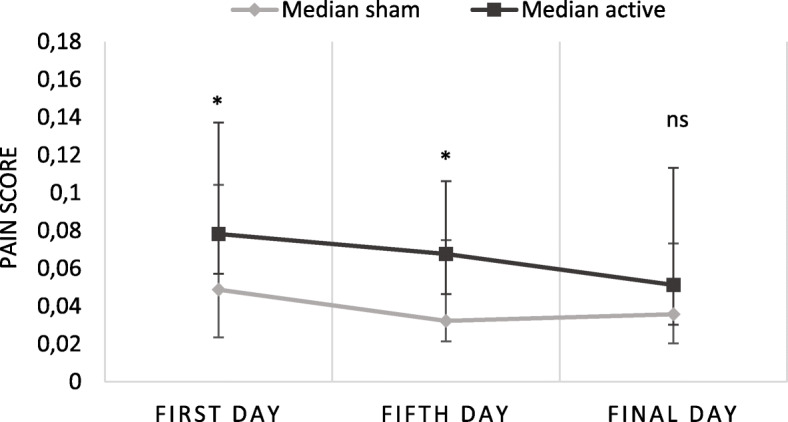
Median of pain rating compensated for titration in the sham and active group in the blind phase, whiskers indicate interquartile range, * indicate *p* < 0.05. N=21/18 on the first and fifth day, and 19/17 on the final day in sham/active groups respectively

The subjective pain score, compensated for titration, was higher in the group receiving active treatment. The pain decreased to a further extent in the active group compared to the group receiving sham treatment (Fig. 1).

An Independent-Samples Mann-Whitney U test showed a statistically significant difference in pain scores between the active (*N* = 18) and the sham (*N* = 21) group on the first (U = 263.5, *p* = 0.035) and fifth day (U = 271.0, *p* = 0.020), but not between the active (*N* = 17) and the sham (*N* = 19) group on the final day (U = 210.5, *p* = 0.121).

A related-samples Friedman’s test of differences among repeated measures was conducted in order to analyze the pain scores over time. When analyzing the pain rating score in the active group (from those with data from all three time points, *N* = 16), there was a statistically significant difference in pain score over time (*χ*^2^(2) = 9.855, *p* = 0.007), with an effect size of w = 0.3. There were significant differences between the first and the fifth day (z = 2.48, *p* = 0.040) and the first and the final day (z = 2.56, *p* = 0.031) with decreasing pain rating scores. There was no significant difference between the fifth and the final day (z = 0.09, *p* = 1.000). In the group receiving sham treatment (*N* = 19), there was a significant difference in pain score over time (*χ*^2^(2) = 6.880, *p* = 0.032). However, when values were adjusted by the Bonferroni correction for multiple tests there was no significant post hoc tests (first-fifth day: z = 1.30, *p* = 0.583, first-final day: z = 2.11, *p* = 0.105, fifth-final day: z = 0.81, *p* = 1.000).

A non-parametric ANCOVA with change in MADRS-S rating as covariate was conducted to control for treatment effect on depressive symptoms. There was no main effect of symptom change (F = 0.733, *p* = 0.398) and no significant time x symptom change interaction (F = 0.135, *p* = 0.716).

Median (IQR) pain ratings, not adjusted for titration of stimulation intensity, for the active/sham group were 4 (2) / 2 (4) on the first day, 3 (2) / 1 (2) on the fifth day, and 2.5 (4) / 2 (2) on the final day, respectively.

#### Open phase

**Fig. 2:**
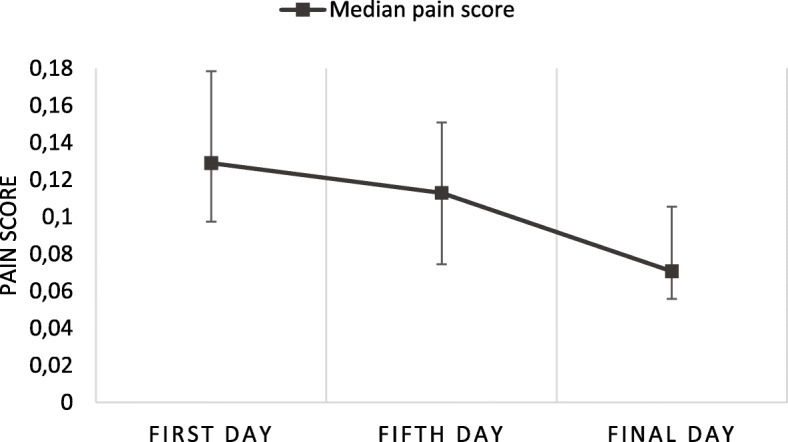
Median of pain rating compensated for titration in the open phase, whiskers indicate interquartile range. *N* = 19 on the first day and *N* = 18 on the fifth and on the final day

The group allocated to sham iTBS received active iTBS in a subsequent open phase. There, the pain ratings were higher in the beginning of treatment, showing a similar trend of decreasing pain as the group receiving active rTMS during the blind phase (Fig. 2). Median (IQR) pain ratings, not adjusted for titration of stimulation intensity, were 5 (2) on the first day, 4 (4) on the fifth day, and 3 (3) on the final day.

A related-samples Friedman’s test of differences among repeated measures comparing pain rating in the open phase (from those with data from all three time points, *N* = 18), showed a significant difference in pain scores over time (*χ*^2^ = 15.129, *p* = 0.001). Dunn-Bonferroni post hoc tests were conducted with adjustments by the Bonferroni correction for multiple tests showing a significant difference between the first and the final day (z = 3.59, p = 0.001) but not between the first and the fifth day (z = 1.42, *p* = 0.470) or the fifth and the final day (z = 2.17, *p* = 0.091).

## Discussion

### Key results

In this first double-blind study of dorsomedial iTBS we observed that the group receiving active iTBS experienced more pain in the beginning of the treatment course compared to sham. The pain ratings decreased significantly over time in the group receiving active iTBS, but not to the same extent in the group receiving sham treatment, indicating an analgesic effect of, or habituation to, dorsomedial iTBS starting early on in the treatment course. During the open phase, when patients who initially received sham treatment later received active iTBS, similar findings were observed.

### Comparison with previous studies and literature

#### Decreasing pain over time

As the present study, to our knowledge, is the first to assess pain rating of dorsomedial iTBS in a double-blinded fashion we can only relate our findings to previous work studying the pain related to the application of conventional rTMS with 10 Hz over the DLPFC. Our results correspond to the study by Borckardt et al. [6], with active iTBS showing a higher overall pain rating compared to sham and the pain rating decreasing over time particularly in the active group. The two studies differ in treatment protocol (high frequency rTMS vs. iTBS) and stimulation target (DLPFC vs. DMPFC), an interesting observation to consider when trying to understand the underlying physiology behind the decreasing pain.

This might be explained by another brain area connected to both DLPFC and DMPFC with an important role in pain processing, i.e. the anterior cingulate cortex (ACC). A meta-analysis exploring the activation and localization of cortical areas involved in pain processing showed that the right ACC had the highest likelihood of being activated when painful stimuli were applied [15]. A study by Bingel et al. (2007) showed that the subgenual region of the ACC (sgACC) was involved in habituation of pain, the decreasing response to a repetitive noxious stimulus [16]. One could speculate whether the decrease in pain in our study could be explained by a connection between DMPFC and the ACC, a region which is indirectly targeted by DLPFC stimulation when treating depression [17].

A study by Fox et al. (2012) suggests that the antidepressant effect of rTMS over the DLPFC may partly be explained by a decreased activity in the sgACC and other limbic regions, as a result of the treatment [18]. Further, another study found that the functional connectivity between DMPFC and the sgACC could predict whether patients would respond to treatment. Patients with higher baseline connectivity between DMPFC and sgACC were more likely to have better treatment response. They also found that the connectivity changed throughout the treatment and that patients responding well to treatment had more anti-correlation between sgACC and DMPFC [19]. In short, there is evidence in the literature linking the ACC to both pain perception and depression, as well as mechanisms underlying its treatment. In our study we used change in MADRS-S rating to control for treatment response. As has been reported in Struckmann et al. (2020), there was a non-significant antidepressive effect on the group level [14]. In our data, we found no significant time x symptom change interaction, indicating that the decreasing pain over time could not be explained by the antidepressant effect of the treatment. However following the above hypothesized sgACC-DMPFC network effect on both pain habituation and depressive symptoms, we would have expected to also find an antidepressant effect. The lack of such can possibly be explained by differences in the time needed for the effects to occur. While the pain habituation occurred rather fast, i.e. already in the first treatment week, more than the targeted ten treatment days might have been necessary for a corresponding clinical antidepressant effect. This argument has also been raised by a recent meta-analysis [20], reporting that an accelerated protocol did not lead to a faster treatment response than a conventional one-daily protocol.

#### Titration as a pain reducing strategy

The fact that patients in our study, especially in the active group, overall received higher intensity of stimulation towards the end of the treatment course, may have affected the pain rating scores. We do not know how a supposed habituation would have affected the pain rating scores if the individually predetermined target magnetic stimulator intensity was applied on the first day without titration. However, one could expect that tolerability would decrease and drop-out rates would probably have been considerably higher due to more discomfort.

For a session to be regarded as a complete treatment session at least 50% of the trains had to reach target treatment intensity. If a session was not complete, the treatment course was prolonged with a maximum of five additional days, which might be a good way to increase tolerability. However, longer treatment courses mean higher costs, potentially limiting accessibility. A pilot study on strategies to reduce rTMS-related pain showed a considerable reduction in pain by injections of lidocaine (with or without epinephrine). They also showed a smaller reduction in rTMS-related pain by applying foam padding between coil and scalp, but no effect by applying topical local anesthetics cream [21]. Studies with larger sample sizes on how to reduce the pain of rTMS without prolonging the treatment would be an interesting area for future research. At the same time, the possible analgesic effect of dorsomedial iTBS might be worthwhile exploring in treatment of various types of pain.

### Strengths and limitations

One of the limitations of this study was the instruments used for pain rating. Patients decided for themselves if they preferred pain rating with VAS or NRS, and therefore both scales were used inconsistently, and without any recording which scale was used. However, the importance of the limitation remains uncertain. A review investigating pain assessment in 54 papers showed that the NRS and VAS scores overall corresponded, with only few exceptions where higher scores were reported with VAS [22]. Another limitation is that we did not collect data on physical pain reactions such as pulse increase or galvanic skin response that might have offered additional biological understanding of the iTBS induced scalp pain apart from subjective ratings. Another limitation of this study was the modest sample size, which limits the statistical power and increases the risk of type II errors.

A strength of our methodology was the study sham-controlled parallel group design and that the pain scores were adjusted for titration in both groups. By doing this, comparisons between active group and sham group, where target intensity was reached to a greater extent, were more accurate than analyzing the original pain score. However, we do not know if the assumption of a linear relation between stimulation intensity and subjective pain rating is met. By allowing inclusion of patients with concomitant diagnoses such as autism spectrum disorders or personality disorder, the patients in this study adequately represented the general clinical patient where multi-morbidity is common, which promotes generalizability of the findings.

## Conclusions

To conclude, active iTBS was more painful than sham in the beginning of the treatment course. The painfulness of active iTBS decreased over time and converged on sham iTBS, with the two being equally tolerable towards the end. When the group allocated to sham iTBS received active iTBS in a subsequent open phase there was a similar trend of decreasing pain. Further research on how to best reduce pain of depression treatment with iTBS could enable faster titration and shorter protocols and increase tolerability.

The outcome of this study can be used clinically to inform patients that during treatment of depression with iTBS, the pain decreases throughout the treatment course.

## Data Availability

We do not have a permit from the ERB to make the data publicly available. The data is available from the corresponding author on reasonable request.

## Abbreviations

rTMS: Repetitive transcranial magnetic stimulation
iTBS: Intermittent theta burst stimulation
DLPFC: Dorsolateral prefrontal cortex
DMPFC: Dorsomedial prefrontal cortex
TBS: Theta burst stimulation
TENS: Transcutaneous electric stimulation
M.I.N.I.: Mini international neuropsychiatric interview
MADRS: Montgomery Asberg Depression Rating Scale
RMT: Resting motor threshold
VAS: Visual analogue scale
NRS: Numeric rating scale
ACC: Anterior cingulate cortex
sgACC: Subgenual anterior cingulate cortex

## References

[1] Lefaucheur J-P, André-Obadia N, Antal A, Ayache SS, Baeken C, Benninger DH (2014). Evidence-based guidelines on the therapeutic use of repetitive transcranial magnetic stimulation (rTMS). Clin Neurophysiol Off J Int Fed Clin Neurophysiol.

[2] Downar J, Daskalakis ZJ (2013). New targets for rTMS in depression: a review of convergent evidence. Brain Stimulat.

[3] Bakker N, Shahab S, Giacobbe P, Blumberger DM, Daskalakis ZJ, Kennedy SH (2015). rTMS of the dorsomedial prefrontal cortex for major depression: safety, tolerability, effectiveness, and outcome predictors for 10 Hz versus intermittent theta-burst stimulation. Brain Stimulat.

[4] Blumberger DM, Vila-Rodriguez F, Thorpe KE, Feffer K, Noda Y, Giacobbe P (2018). Effectiveness of theta burst versus high-frequency repetitive transcranial magnetic stimulation in patients with depression (THREE-D): a randomised non-inferiority trial. Lancet.

[5] Janicak PG, Dokucu ME (2015). Transcranial magnetic stimulation for the treatment of major depression. Neuropsychiatr Dis Treat.

[6] Borckardt JJ, Nahas ZH, Teal J, Lisanby SH, McDonald WM, Avery D (2013). The painfulness of active, but not sham, transcranial magnetic stimulation decreases rapidly over time: results from the double-blind phase of the OPT-TMS trial. Brain Stimulat.

[7] Pettersson A, Modin S, Wahlström R, af Winklerfelt Hammarberg S, Krakau I (2018). The mini-international neuropsychiatric interview is useful and well accepted as part of the clinical assessment for depression and anxiety in primary care: a mixed-methods study. BMC Fam Pract.

[8] Llerena K, Park SG, McCarthy JM, Couture SM, Bennett ME, Blanchard JJ (2013). The motivation and pleasure scale-self-report (MAP-SR): reliability and validity of a self-report measure of negative symptoms. Compr Psychiatry.

[9] Svanborg P, Asberg M (2001). A comparison between the Beck depression inventory (BDI) and the self-rating version of the Montgomery Asberg depression rating scale (MADRS). J Affect Disord.

[10] Motor Threshold Assessment Tool 2.0. https://www.clinicalresearcher.org/software.htm. Accessed 5 Nov 2019.

[11] Mishory A, Molnar C, Koola J, Li X, Kozel FA, Myrick H (2004). The maximum-likelihood strategy for determining transcranial magnetic stimulation motor threshold, using parameter estimation by sequential testing is faster than conventional methods with similar precision. J ECT.

[12] Mir-Moghtadaei A, Giacobbe P, Daskalakis ZJ, Blumberger DM, Downar J (2016). Validation of a 25% Nasion-inion heuristic for locating the dorsomedial prefrontal cortex for repetitive transcranial magnetic stimulation. Brain Stimulat.

[13] Opitz A, Legon W, Mueller J, Barbour A, Paulus W, Tyler WJ (2014). Is sham cTBS real cTBS? The effect on EEG dynamics. Front Hum Neurosci.

[14] Struckmann W, Persson J, Weigl W, Gingnell M, Bodén R (2020). Modulation of the prefrontal blood oxygenation response to intermittent theta-burst stimulation in depression: a sham-controlled study with functional near-infrared spectroscopy. World J Biol Psychiatry.

[15] Duerden EG, Albanese M-C (2013). Localization of pain-related brain activation: a meta-analysis of neuroimaging data. Hum Brain Mapp.

[16] Bingel U, Schoell E, Herken W, Büchel C, May A (2007). Habituation to painful stimulation involves the antinociceptive system. Pain..

[17] Weigand A, Horn A, Caballero R, Cooke D, Stern AP, Taylor SF (2018). Prospective validation that Subgenual connectivity predicts antidepressant efficacy of transcranial magnetic stimulation sites. Biol Psychiatry.

[18] Fox MD, Buckner RL, White MP, Greicius MD, Pascual-Leone A (2012). Efficacy of transcranial magnetic stimulation targets for depression is related to intrinsic functional connectivity with the subgenual cingulate. Biol Psychiatry.

[19] Salomons TV, Dunlop K, Kennedy SH, Flint A, Geraci J, Giacobbe P (2014). Resting-state cortico-thalamic-striatal connectivity predicts response to dorsomedial prefrontal rTMS in major depressive disorder. Neuropsychopharmacol Off Publ Am Coll Neuropsychopharmacol.

[20] Kaster TS, Chen L, Daskalakis ZJ, Hoy KE, Blumberger DM, Fitzgerald PB (2020). Depressive symptom trajectories associated with standard and accelerated rTMS. Brain Stimulat.

[21] Borckardt JJ, Smith AR, Hutcheson K, Johnson K, Nahas Z, Anderson B (2006). Reducing pain and unpleasantness during repetitive transcranial magnetic stimulation. J ECT.

[22] Hjermstad MJ, Fayers PM, Haugen DF, Caraceni A, Hanks GW, Loge JH (2011). Studies comparing numerical rating scales, verbal rating scales, and visual analogue scales for assessment of pain intensity in adults: a systematic literature review. J Pain Symptom Manag.

